# Comparison of Optical Properties and Fracture Loads of Multilayer Monolithic Zirconia Crowns with Different Yttria Levels

**DOI:** 10.3390/jfb15080228

**Published:** 2024-08-16

**Authors:** Chien-Ming Kang, Tzu-Yu Peng, Yan-An Wu, Chi-Fei Hsieh, Miao-Ching Chi, Hsuan-Yu Wu, Zih-Chan Lin

**Affiliations:** 1 Huayi Dental Laboratory, Taipei 10491, Taiwan; kjm670815@hotmail.com; 2 School of Dentistry, College of Oral Medicine, Taipei Medical University, Taipei 11031, Taiwan; 3 Research Center of Digital Oral Science and Technology, College of Oral Medicine, Taipei Medical University, Taipei 11031, Taiwan; 4 School of Dental Technology, College of Oral Medicine, Taipei Medical University, Taipei 11031, Taiwan; 5 Department of Respiratory Care, Chang Gung University of Science and Technology, Chiayi 61363, Taiwan; 6 Chronic Diseases and Health Promotion Research Center, Chang Gung University of Science and Technology, Chiayi 61363, Taiwan; 7 Division of Integrated Health Sciences, Graduate School of Biomedical and Health Sciences, Hiroshima University, Hiroshima 834-8553, Japan

**Keywords:** multilayer monolithic zirconia, yttria stabilizer, dental crown, fracture loads, translucency, color accuracy, digital colorimeter, CAD/CAM

## Abstract

Multilayer monolithic zirconia, which incorporates polychromatic layers that mimic natural tooth gradients, offers enhanced aesthetics and functionality while reducing debonding risks and improving fabrication efficiency. However, uncertainties remain regarding the fracture characteristics of multilayer monolithic zirconia crowns under occlusal loading, whether composed of uniform or combined yttria levels. The current study investigated how variations in yttria levels and thicknesses affected the optical properties and fracture loads of multilayer monolithic zirconia. Samples of multilayer monolithic zirconia in the Vita A1 shade were used, while employing 3Y (SZ) and 4Y + 5Y (AZ) yttria levels. The optical properties, including the color difference (Δ*E_WS_*) and translucency parameters (*TP*_00_), were measured using a digital colorimeter. The fracture loads were analyzed using a universal testing machine, and fractured surfaces were examined under a stereomicroscope. Statistical analyses assessed the impacts of the yttria levels and sample thicknesses on the optical properties. The Δ*E_WS_* values of SZ ranged 3.6 to 4.0, while for AZ, Δ*E_WS_* at 0.5 mm was 3.9 and <2.6 for other thicknesses. The *TP*_00_ values decreased with an increased thickness, with AZ generally exhibiting greater translucency than SZ. In the fracture load investigations, SZ (>1600 N) generally exceeded AZ (>1260 N), with fracture loads notably increasing with thickness, particularly for premolars (SZ > 3270 N, AZ > 2257 N). SZ predominantly exhibited partial and complete fractures, whereas AZ showed fewer non-fracture categorizations. Complete fractures began with dense, irregular cracks that extended outward to reveal smooth surfaces, while premolars subjected to higher loads exhibited concentric ripple-like structures. Partial fractures revealed radial textures indicative of areas of stress concentration. In summary, higher yttria levels were correlated with increased translucency, while variations in the fracture loads primarily stemmed from differences in the tooth position or thickness. Overall, multilayer monolithic zirconia incorporating combined yttria levels of 4Y + 5Y (AZ) offered high translucency, precise color matching, and substantial fracture resistance, rendering it highly suitable for aesthetic and functional dental applications.

## 1. Introduction

Multilayer monolithic zirconia achieves consistent and stable coloration through a production process involving multiple polychromatic layers that mimic the natural shade gradient of teeth. Compared to traditional zirconia, this approach reduces potential operator errors in staining, resulting in superior aesthetic outcomes of final restorations [1,2,3,4]. Monolithic zirconia offers excellent functionality and aesthetics while requiring minimal tooth preparation, thereby reducing the risk of structural damage and preparation trauma [5,6]. Survival rates of multilayer monolithic zirconia range from 91% to 100% over follow-up periods of 0.3–2.1 years [6], while eliminating the need for a veneer layer for color adjustment, which minimizes risks of chipping and debonding and enhances fabrication efficiency [2,7].

Zirconia green bodies consist primarily of high-purity zirconia powder [4], with added metal oxides, such as MgO, Al_2_O_3_, and Y_2_O_3_, to enhance toughness and stability [8,9]. The most commonly used is 3 mol% yttria-stabilized tetragonal zirconia polycrystal (3Y-TZP) [10], with the final shade adjustment achieved by incorporating the desired colorants [11]. In multilayer monolithic zirconia, the color gradient significantly depends on the choices of stabilizers and colorants. For monolithic zirconia stabilized with a uniform yttria level (e.g., 3Y), the color gradient results from varying the shade of each layer [8]; yet, this type of multilayer monolithic zirconia provides only a color gradient and lacks variations in translucency. The latest generation of multilayer monolithic zirconia employs combinations of different yttria mol% levels, such as 3Y-TZP combined with 5 mol% partially stabilized zirconia (5Y-PSZ), or a combination of 4 and 5 mol% partially stabilized zirconia (4Y + 5Y), and these are commonly observed in clinical practice [7]. These achieve both color gradients and variations in translucency and strength, thereby expanding the aesthetic potential of zirconia [12].

Researchers have investigated the optical properties and color accuracy of multilayer monolithic zirconia, including different yttria combinations (e.g., 3Y + 5Y, 4Y + 5Y, 5Y, etc.). Kang et al. demonstrated that the 4Y + 5Y combination exhibited superior performance in terms of color accuracy and aesthetics [7]. Regarding the influence of thickness on optical properties, Tabatabaian et al. [13] and Kang et al. [14] separately studied the final colors of monolithic zirconia and multilayer monolithic zirconia, confirming that the optimal thickness for color accuracy was at least 1.0 mm. Despite consistent findings across those studies, showing that a sufficient thickness enables multilayer monolithic zirconia to demonstrate excellent optical performance and fracture resistance and withstand occlusal loads [15,16], most of those studies were limited to testing samples such as discs or rod shapes. Whether multilayer monolithic zirconia maintains its fracture resistance under occlusal loading when fabricated into crown morphologies remains to be further investigated. The authors previously evaluated fracture loads of crown-shaped specimens made from 4Y + 5Y multilayer monolithic zirconia, revealing that thicknesses exceeding 1.0 mm ensured adequate mechanical properties [17]. However, differences in the fracture loads and optical properties among samples of multilayer monolithic zirconia with different yttria levels, whether uniform or combined to form color gradients, have not been fully elucidated. Therefore, in the current study, we addressed the existing research gaps by investigating the effects of yttria levels on the fracture loads and optical properties of multilayer monolithic zirconia.

## 2. Materials and Methods

### 2.1. Sample Preparation

The samples used in this experiment included plate-shaped and crown-shaped multilayer monolithic zirconia, as well as metal abutments. Two types of multilayer monolithic zirconia in the Vita A1 shade were used, each composed of different yttria levels: one was uniformly composed of 3Y with 4.5 to 5.5 wt.% of Y_2_O_3_ (Superfectzir; Aidite Technology, Qinhuangdao, China; denoted as SZ), and the other was a combination of 4Y and 5Y with 4.0 to 10.0 wt.% of Y_2_O_3_ (Aizir; Aidite Technology; denoted as AZ). All samples were prepared using a dental computer-assisted design (CAD)/computer-assisted manufacturing (CAM) system (Cameo 250i; Aidite Technology), following a method described by Kang et al. [17]. The multilayer monolithic zirconia plate samples (*n* = 15) used for optical testing had dimensions of 10 mm × 10 mm and came in four thicknesses: 0.5, 1.0, 1.5, and 2.0 mm. The metal abutments and multilayer monolithic zirconia crown samples were prepared for three tooth positions: maxillary central incisor, maxillary first premolar, and mandibular first molar. Meanwhile, the multilayer monolithic zirconia crown samples (*n* = 15) were designed with three thicknesses for each tooth position: 1.0, 1.5, and 2.0 mm. After preparation, the samples were cleaned using an ultrasonic cleaner with alcohol and isopropanol for 15 min, and then left to completely dry at room temperature.

### 2.2. Optical Property Analyses

The test samples were placed on professional white and black photography cards (QP cards 101; QPcard, Helsingborg, Sweden). A digital colorimeter (OptiShade StyleItaliano; Smile Line, St-Imier, Switzerland) was then used to capture the colors of the samples. The colors were recorded based on the Commission Internationale de l’Eclairage (CIE) system and quantified using the color attributes L* (lightness), a* (red–green), and b* (yellow–blue). The measurements were consistently performed under the same light source to eliminate potential variables, and the colorimeter was recalibrated before each test.

To confirm the color accuracy of the samples, the color attributes measured on a white substrate (Δ*E_WS_*) were calculated and compared to the A1 Vita shade guide using the following formula:

$${\Delta E}_{ws}=\sqrt{{\left(\frac{\Delta L^{\prime }}{k_{L}{\;S}_{L}}\right)}^{2}+{\left(\frac{\Delta C^{\prime }}{k_{C}{\;S}_{C}}\right)}^{2}+{\left(\frac{\Delta H^{\prime }}{k_{H}{\;S}_{H}}\right)}^{2}+R_{T}\left(\frac{\Delta C^{\prime }}{k_{C}{\;S}_{C}}\right)\left(\frac{\Delta H^{\prime }}{k_{H}{\;S}_{H}}\right)\;}$$

where, Δ*L′*, Δ*C′*, and Δ*H′* are the differences in the lightness, chroma, and hue, respectively; *k_L_*, *k_C_*, and *k_H_* are the weighting coefficients; *S_L_*, *S_C_*, and *S_H_* are the mean values; and *R_T_* is the overall correction coefficient for the chroma and hue differences.

The translucency parameter (*TP*_00_) of the plated samples was measured to accurately assess the translucency of the multilayer monolithic zirconia. The analysis was conducted using both the three-third (incisal, body, and cervical), and the nine-square division methods described by Kang et al. [17]. Finally, the *TP*_00_ value was calculated from the obtained color attributes (*n* = 15) using the following formula:

$${TP}_{00}=\sqrt{{\;\left(\frac{L_{B}^{\prime }-L_{W}^{\prime }}{k_{L}{\;S}_{L}}\right)}^{2}+{\left(\frac{C_{B}^{\prime }-C_{W}^{\prime }}{k_{C}{\;S}_{C}}\right)}^{2}+{\left(\frac{H_{B}^{\prime }-H_{W}^{\prime }}{k_{H}{\;S}_{H}}\right)}^{2}+R_{T}\left(\frac{C_{B}^{\prime }-C_{W}^{\prime }}{k_{C}{\;S}_{C}}\right)\left(\frac{H_{B}^{\prime }-H_{W}^{\prime }}{k_{H}{\;S}_{H}}\right)\;}$$

where *L′_B_*, *C′_B_*, and *H′_B_* are the lightness, chroma, and hue of the samples on a black background; and *L′_W_*, *C′_W_*, and *H′_W_* are the lightness, chroma, and hue of the samples on a white background, respectively.

### 2.3. Fracture Load Testing

**Figure 1:**
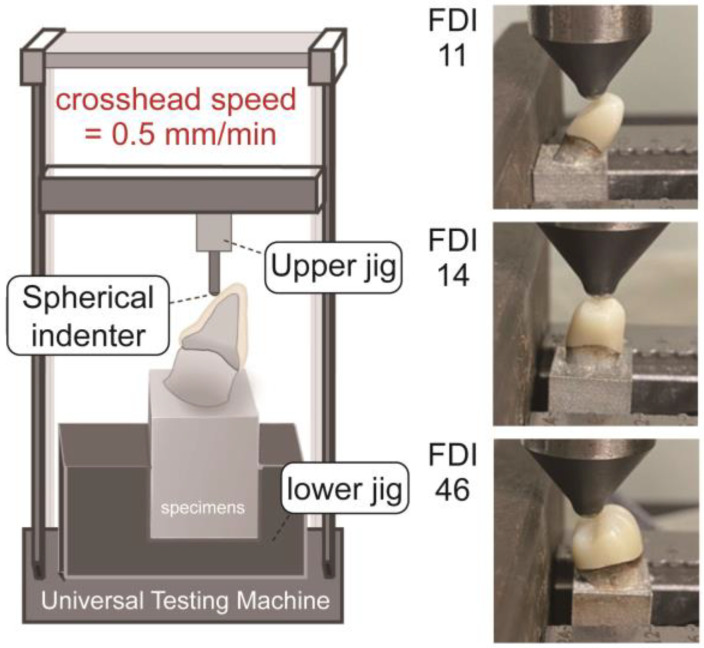
Experimental set-up of the fracture load testing.

To avoid variations due to manual processing, no polishing or glazing was performed on the multilayer monolithic zirconia crowns in this experiment. All specimens were bonded to metal abutments using resin cement (RelyX™ U200; 3M EPSE, St. Paul, MN, USA). After removing the excess cement, a vertical force of 4.9 N was applied to the crowns, which were then light-cured and left to stand for 1 h to ensure complete curing. Fracture load testing was conducted using a universal testing machine (AGX-V; Shimadzu, Kyoto, Japan). The metal abutment was fixed in the lower jig, and a spherical indenter with a radius of 2.5 mm was used in the upper jig to contact the crown in the tripod occlusion mode, applying stress at a crosshead speed of 0.5 mm/min, as shown in Figure 1. Stress was applied starting from 0 until a stress drop exceeding 10 N appeared on the stress–strain curve, indicating crown fracture, at which point the experiment was terminated. The load (N) at the time of fracture was immediately recorded, and the fractured surface morphology was observed with a dental microscope and categorized as no fracture, crack, partial fracture, or complete fracture. Representative crown samples were selected for image analysis using an automatic focus-stacking function-equipped stereomicroscope for microphotography (TORI FOCUS, Taiwan Ocean Research Institute, Kaohsiung, Taiwan) to detail the fracture initiation.

### 2.4. Statistical Analysis

All data in the current study are presented as the mean ± standard deviation (SD). A normality analysis, initially conducted using the Shapiro–Wilk test, confirmed that the experimental data were normally distributed, allowing for the subsequent use of parametric analyses. A Pearson correlation analysis, one-way analysis of variance (ANOVA), and two-way ANOVA were performed to statistically investigate the effects of yttria levels and thicknesses on the optical properties and fracture loads. Post hoc comparisons among the groups were conducted using Tukey’s honest significant difference (HSD) test. Statistical analyses were carried out using SPSS (v19; IBM, Armonk, NY, USA), with the significance level set to 5%.

## 3. Results

### 3.1. Color Accuracy and Translucency

**Figure 2:**
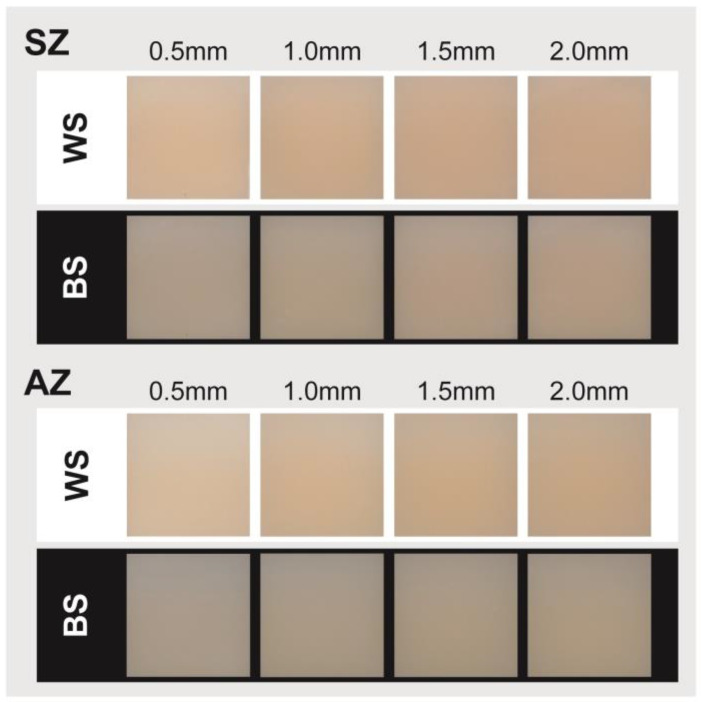
Color appearances of plate-shaped test samples. Two multilayer monolithic zirconia (SZ and AZ) plate-shaped (10 mm × 10 mm) test samples of various thicknesses (0.5, 1.0, 1.5, and 2.0 mm), measured with a digital colorimeter under white (WS) and black (BS) substrates.

**Figure 3:**
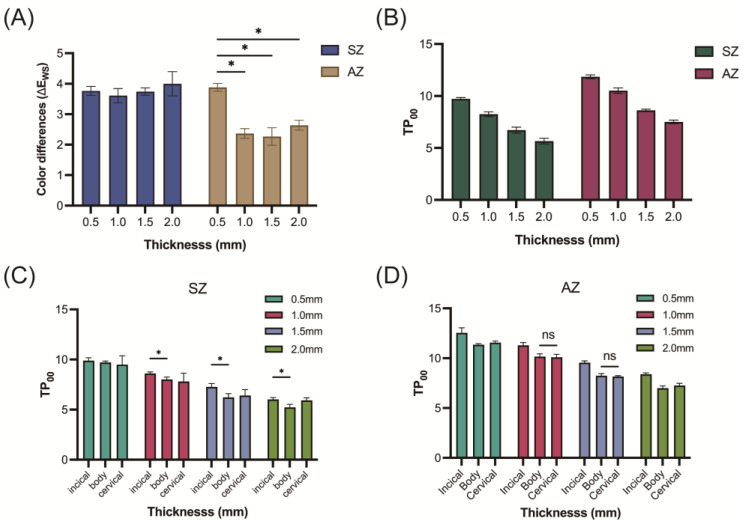
Optical analysis results. Color differences (Δ*E_WS_*) (**A**) and translucency parameter (*TP*_00_) (**B**) results of two multilayer monolithic zirconia (SZ and AZ) plate-shaped (10 mm × 10 mm) samples of various thicknesses (0.5, 1.0, 1.5, and 2.0 mm) under the nine-square division method. Note that the Δ*E_WS_* was calculated based on the color attributes measured on a white substrate and compared with the A1 Vita shade guide. The *TP*_00_ results of SZ (**C**) and AZ (**D**) under the three-third division method. Asterisks (*) and ns respectively indicate a statistically significant difference (*p* < 0.05) or not (*p* > 0.05) between the groups.

Figure 2 illustrates the color appearances of the plate-shaped zirconia samples on white (WS) and black (BS) substrates using a QP card. CIE L*, a*, b*, Δ*E*, and *TP*_00_ values are presented in Table 1 and Figure 3. Both the SZ and AZ samples exhibited higher lightness on the WS compared to the BS, with significantly higher L* values observed for the 0.5 mm thickness (*p* < 0.05). In terms of color differences (Δ*E_WS_*) (Table 1, Figure 3A), SZ showed values ranging from 3.6 to 4.0, with no significant differences among the thicknesses (*p* > 0.05), and for AZ, the Δ*E_WS_* at 0.5 mm was 3.9, significantly higher than for the other thicknesses (*p* < 0.05), whereas the other thicknesses remained below 2.6, with no significant differences (*p* > 0.05).

Translucency, assessed via the nine-square division method (Table 1, Figure 3B), was analyzed using Pearson correlations (Table 2), revealing a significant negative correlation (*r* < −0.99, *p* < 0.05) with an increasing thickness. Further analysis using the three-third division method (Figure 3C,D) indicated a significant decrease in *TP*_00_ with an increasing thickness across all layers (incisal, body, and cervical) (*p* < 0.05). The correlation analysis (Table 2) showed that SZ, with lower yttria levels (3Y), exhibited significantly lower *TP*_00_ at the same thickness compared to AZ (4Y + 5Y), indicating a positive correlation between the yttria levels and *TP*_00_ (*r* = 1.00, *p* < 0.05).

### 3.2. Fracture Loads

**Figure 4:**
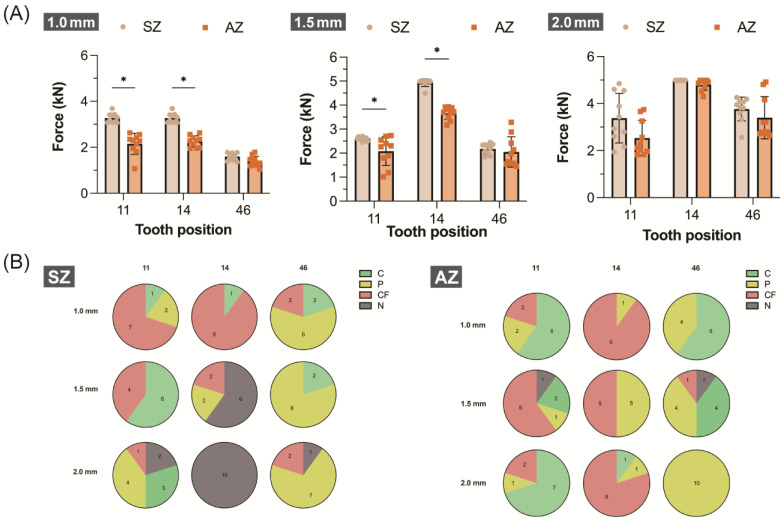
Fracture strength results. (**A**) Load values (N) at fracture of two multilayer monolithic zirconia (SZ and AZ) crown samples under three different thicknesses (1.0, 1.5, and 2.0 mm) and three tooth positions (11, maxillary right central incisor; 14, maxillary right first premolar; and 46, mandibular right first molar). Asterisks (*) indicate statistically significant differences among the groups (*p* < 0.05). (**B**) Optical microscopic categorization and counting of fractured samples of SZ and AZ, classified as crack (C), partial fracture (P), complete fracture (CF), and no fracture (N).

Comparing the influence of different yttria levels on the fracture load, SZ generally demonstrated higher values than AZ (Table 3). Tukey’s HSD post hoc test (Figure 4A) revealed no significant differences between SZ and AZ except at a 1.0 mm thickness for the incisors (*p* > 0.05). Significant differences were observed for the premolars, except at a 2.0 mm thickness (*p* < 0.05), while no significant differences were found for the molars across all thicknesses (*p* > 0.05). Regardless of the yttria levels (Table 4), the fracture load increased with the thickness at the same tooth position, showing a significant positive correlation (*r* > 0.57, *p* < 0.05). The premolars consistently exhibited the highest fracture loads (SZ > 3270 N, AZ > 2257 N) across all thicknesses (Table 3). In SZ, except at a 2.0 mm thickness, the incisors displayed significantly greater fracture loads than the molars (*p* < 0.05). For AZ, there was no load difference between the molars and incisors, except at a 2.0 mm thickness (*p* > 0.05).

**Figure 5:**
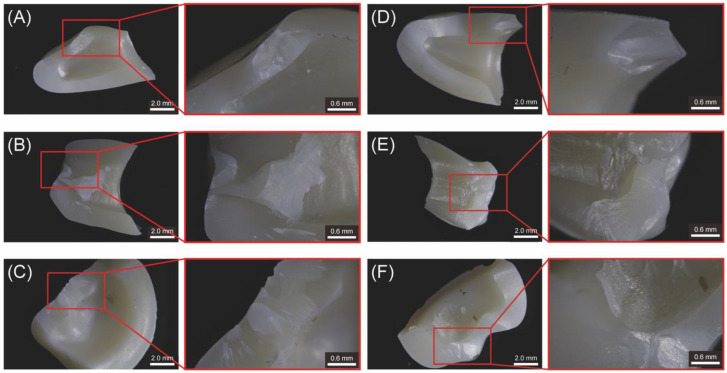
Observation of representative fracture surface structures of two multilayer monolithic zirconia crown samples (SZ and AZ) using an automatic focus-stacking function stereomicroscope. (**A**–**C**) show SZ samples, while (**D**–**F**) show AZ samples, corresponding to a maxillary right central incisor (**A**,**D**), maxillary right first premolar (**B**,**E**), and mandibular right first molar (**C**,**F**), respectively.

The results of the fracture surface categorization for the crown-shaped zirconia samples are presented in Table 3 and Figure 5. In SZ, partial fracture and complete fracture were predominant, particularly in the premolars with thicknesses of 1.5 and 2.0 mm. AZ showed fewer instances of no fractures, with similar frequencies across the other three fracture classifications. Complete fractures (Figure 5A,B,D,F) exhibited initial cracks with dense, irregular structures appearing light, extending outward to reveal smooth surfaces at the incisor (Figure 5A) and marginal cracking at the AZ (Figure 5D). In the SZ premolars (Figure 5B), which experienced significantly higher fracture loads (>3270 N, *p* < 0.05), concentric ripple-like structures extended from the crack initiation point. Partial fractures (Figure 5C–E) exhibited dense, irregular structures with radial textures as the cracks propagated, highlighting similar stress concentration areas.

## 4. Discussion

Zirconia is a metastable polycrystalline ceramic composed of monoclinic, tetragonal, and cubic phases, with the phase transitions occurring at different temperatures. Among these, the tetragonal phase, which exhibits the best mechanical strength, is maintained at room temperature with the addition of oxide stabilizers, most notably 3Y [8,9,10]. Research by Kim et al. indicated that increasing the yttria levels, such as 4Y or 5Y, led to a higher proportion of the cubic phase, reducing light scattering at the grain boundaries and thereby increasing translucency, making zirconia suitable for aesthetic applications [18]. However, research suggested that high translucency in monolithic multilayer pre-colored zirconia can result in color distortions [7], and the increased cubic phase caused by a higher yttria level may decrease the fracture load [19]. Therefore, balancing yttria levels is a significant challenge in the use of monolithic multilayer pre-colored zirconia.

When using monolithic multilayer pre-colored zirconia, it is essential to assess the restoration thickness. Tabatabaian et al. reported that the minimum thickness of monolithic zirconia should be 0.9 mm to achieve an acceptable final color [13], whereas previous research suggested that the minimum required thickness for zirconia crowns or veneer restorations was 1.5 mm [20,21]. Therefore, in the current study, four thicknesses (0.5, 1.0, 1.5, and 2.0 mm) on two types of substrates (WS and BS) were included (Figure 2). The results of the optical properties of the two materials (SZ and AZ) significantly differed (Table 1). SZ, formed by stacking uniform yttria levels with only a color gradient and no transparency gradient, showed no significant differences in its Δ*E_WS_* from the Vita A1 shade guide under different thickness conditions (Figure 3A). In contrast, AZ, which was composed of combined 4Y + 5Y and had both a color gradient and a transparency gradient, exhibited lower *TP*_00_ values than SZ (Figure 3B). However, the *TP*_00_ values for the different sections (incisal, body, or cervical) showed no significant differences, except for slightly better translucency in the incisal region (Figure 3C).

AZ demonstrated higher translucency at 0.5 mm, leading to a significantly higher color difference compared to the other thicknesses (Figure 3A). At 1.0 and 1.5 mm, the Δ*E_WS_* was <2.4, similar to the values reported in the previous literature using the same yttria levels of 4Y + 5Y (1.84–3.09) [7]. Studies indicated that excessive thickness can result in lower light transmission, leading to higher Δ*E* and color deviations [14]. Despite the good translucency of AZ, the *TP*_00_ at the incisal section was significantly higher than those at the body and cervical sections, although there were no significant differences between the body and cervical sections at 1.0 and 1.5 mm thicknesses (Figure 3D). Notably, AZ exhibited consistent Δ*E_WS_* values at 1.0, 1.5, and 2.0 mm, verifying the color accuracy of AZ.

Evaluating the fracture strength is essential to understanding the lifetime of monolithic multilayer pre-colored zirconia [22,23]. Previous research confirmed that the fracture strength of these crowns is influenced by both the tooth position and thickness, with thickness having a more significant impact [17]. While the primary aim of the current study was to investigate the influence of yttria levels on fracture loads, the thickness was also considered a variable. The experimental results demonstrate that the fracture load of the zirconia crowns increased with the thickness in all tooth positions (Table 3). However, when the thickness reached 2.0 mm, the crown morphology began to show excessive bulging, with indistinct separation between the cusps and grooves. Therefore, it is crucial to select a minimum thickness that provides sufficient strength to balance both the aesthetic morphology and mechanical integrity.

SZ, with a uniform yttria level of 3Y, exhibited a greater fracture load than AZ, which contained mixed yttria levels of 4Y and 5Y, regardless of the thickness or tooth position. This difference was attributed to the inverse relationship between the yttria levels and fracture resistance; higher yttria levels result in decreased fracture resistance [24]. Badr et al. supported this finding, demonstrating that the fracture resistance of zirconia is primarily determined by the yttria levels at the occlusal surface, with resistance inversely proportional to those levels [25]. Additionally, the premolars exhibited significantly higher fracture loads compared to the incisors or molars (Figure 4, Table 3). This can be attributed to their morphological structure. Premolars, with only two cusp tips, allow the spherical indenter to be securely located in the central fossa. In contrast, incisors have a labial fossa on the lingual surface, and molars have five cusp tips, both of which can cause the indenter to slip during testing, resulting in lateral forces that may damage the structure [17].

The analysis of fracture loads and surface categories (Figure 4) revealed that the SZ premolars consistently demonstrated the highest fracture load across all test groups at both 1.5 and 2.0 mm. This finding was, however, limited by the 5 kN capacity of the universal testing machine, which constrained measurements beyond this load. At 2.0 mm, nearly all groups surpassed this load limit, resulting in comparable values, reduced SDs, and a 100% incidence of no fracture. Indergård et al. reported that the fracture load range for 3Y zirconia was 3873–7500 N, which exceeded that of 5Y zirconia (2100–4948 N), supporting the findings of the present study [26]. To our knowledge, most masticatory or occlusal stresses are concentrated in the posterior region, where average physiological stresses range from 700 to 1000 N [16,27]. In the current study, both SZ and AZ demonstrated fracture loads exceeding 1260 N, indicating their capability to withstand typical physiological masticatory or occlusal forces, even in anterior positions. Kim et al. reported that inadequate thickness in monolithic zirconia crowns can compromise margin strength, with margin thicknesses below 0.8 mm reducing fracture resistance [16]. Badr et al. found that for monolithic and multi-yttria-layered zirconia crowns, the minimum recommended thicknesses for uniform yttria levels of 3Y, 4Y, and 5Y were 1.0, 1.2, and 1.5 mm, respectively [25]. Based on the current study and related literature, it is recommended that, irrespective of whether the yttria level is 3Y or 4Y + 5Y in monolithic multilayer pre-colored zirconia, a minimum thickness of 1.0 mm should be maintained to ensure both optimal optical performance and adequate fracture resistance.

This experiment was conducted at room temperature without incorporating artificial aging tests, such as thermocycling or thermo-mechanical loading. While previous studies suggested that artificial aging does not influence the fracture resistance of zirconia crowns [25], these factors should be addressed in future research. Furthermore, the fracture load testing in this study employed a single-directional mode with linear up-and-down movement. Future investigations should utilize masticatory simulation systems to better replicate physiological occlusal conditions.

## 5. Conclusions

Based on the findings of this in vitro study, the following conclusions can be drawn:

1. An increased yttria level was associated with enhanced translucency. The multilayer monolithic zirconia crown containing a 4Y + 5Y (AZ) exhibited superior color accuracy compared to that containing only 3Y (SZ), which showed notable color deviations.
2. Variations in the fracture loads were primarily attributed to differences in the tooth position or thickness. Although AZ demonstrated lower fracture loads than SZ, it still showed sufficient values (>1260 N) to withstand biting or occlusion forces.
3. When selecting multilayer monolithic zirconia with higher or combined yttria levels, ensuring a minimum thickness of 1.0 mm is necessary to meet both aesthetic and functional requirements in dental applications.

## Figures and Tables

**Table 1:** Color attributes (L*, a*, b*), color differences, and translucency parameters.

Thickness		0.5 mm			1.0 mm			1.5 mm			2.0 mm		
Color Attributes		L*	a*	b*	L*	a*	b*	L*	a*	b*	L*	a*	b*
SZ	WS	81.0 ± 0.4	2.8 ± 0.5	16.1 ± 1.0	77.4 ± 0.5	3.6 ± 0.4	18.6 ± 0.2	76.0 ± 0.6	4.1 ± 0.2	18.0 ± 0.2	74.7 ± 0.5	4.1 ± 0.4	17.9 ± 0.5
	BS	69.4 ± 0.1	0.5 ± 0.1	9.6 ± 0.3	68.8 ± 0.3	0.6 ± 0.3	11.8 ± 0.2	69.3 ± 0.3	1.5 ± 0.2	12.0 ± 0.2	69.3 ± 0.5	1.5 ± 0.4	13.0 ± 0.3
	Δ*E_WS_*	3.8 ± 0.1			3.6 ± 0.2			3.7 ± 0.1			4.0 ± 0.4		
	*TP*_00_	9.7 ± 0.1			8.2 ± 0.2			6.7 ± 0.3			5.7 ± 0.3		
AZ	WS	82.2 ± 0.2	1.1 ± 0.3	13.6 ± 0.3	79.2 ± 0.5	2.0 ± 0.1	16.7 ± 0.3	75.7 ± 0.2	2.1 ± 0.1	17.9 ± 0.5	74.3 ± 0.1	2.2 ± 0.2	17.6 ± 0.3
	BS	67.7 ± 0.2	−1.0 ± 0.3	7.0 ± 0.2	67.2 ± 0.1	−0.8 ± 0.1	9.8 ± 0.3	66.6 ± 0.1	−0.7 ± 0.1	11.5 ± 0.4	66.7 ± 0.2	−0.5 ± 0.1	11.8 ± 0.4
	Δ*E_WS_*	3.9 ± 0.1			2.4 ± 0.2			2.3 ± 0.2			2.6 ± 0.2		
	*TP*_00_	11.9 ± 0.2			10.5 ± 0.3			8.6 ± 0.1			7.5 ± 0.2		

Color attributes of L* (lightness), a* (red–green), and b* (yellow–blue). WS, white substrate (white QP card); BS, black substrate (black QP card). Color difference (Δ*E_WS_*) was calculated based on the color attributes measured on a white substrate and compared with the A1 Vita shade guide. Translucency parameter (*TP*_00_) was calculated based on the color attributes measured between the black and white substrates.

**Table 2:** Pearson correlation results of *TP*_00_ with different thicknesses and yttria levels.

	Thickness								Yttria Levels
	Three-Third Division						Nine-Square Division		
	Incisal		Body		Cervical				
Zirconia type	SZ	AZ	SZ	AZ	SZ	AZ	SZ	AZ	
Pearson *r* value	−1.000 *	−0.997 *	−0.993 *	−0.995 *	−0.986 *	−0.992 *	−0.997 *	−0.995 *	1.000 *
*p* value	>0.001	0.003	0.007	0.005	0.014	0.008	0.003	0.005	>0.001

* Correlation is significant at the 0.01 level (2-tailed).

**Table 3:** Fracture loads and categories.

		Load at Fracture (Fracture Surface Category)		
Tooth Position		11	14	46
SZ	1.0 mm	2309.03 ± 405.63 (0/1/2/7)	3270.86 ± 206.13 (0/1/0/9)	1600.75 ± 162.72 (0/2/6/2)
	1.5 mm	2609.46 ± 77.14 (0/6/4/0)	4935.91 ± 157.05 (6/0/2/2)	2175.71 ± 218.64 (0/2/0/8)
	2.0 mm	3378.27 ± 1054.11 (2/3/4/1)	4992.05 ± 0.33 (10/0/0/0)	3768.86 ± 506.69 (1/0/2/7)
AZ	1.0 mm	1261.75 ± 366.14 (0/6/2/2)	2257.66 ± 245.96 (0/0/2/8)	1377.75 ± 231.48 (0/6/4/0)
	1.5 mm	2083.85 ± 602.87 (1/2/1/6)	3664.29 ± 260.35 (0/0/5/5)	2048.25 ± 632.40 (1/4/4/1)
	2.0 mm	2533.40 ± 746.91 (0/7/1/2)	4802.90 ± 234.44 (0/1/1/8)	3397.52 ± 894.09 (0/0/10/0)

The load at fracture is presented as the mean ± SD, with the unit being Newtons. The fracture surface category is presented as a count, with the order in parentheses being no fracture/crack/partial fracture/complete fracture. The tooth positions are as follows: 11, maxillary right central incisor; 14, maxillary right first premolar; 46, mandibular right first molar.

**Table 4:** Pearson correlation results of fracture loads with different thicknesses and yttria levels.

	Thickness						Yttria Level								
	Maxillary Right Central Incisor		Maxillary Right First Premolar		Mandibular Right First Molar		Maxillary Right Central incisor			Maxillary Right First Premolar			Mandibular Right First Molar		
	SZ	AZ	SZ	AZ	SZ	AZ	1.0 mm	1.5 mm	2.0 mm	1.0 mm	1.5 mm	2.0 mm	1.0 mm	1.5 mm	2.0 mm
Pearson *r* value	0.570 *	0.674 *	0.866 *	0.974 *	0.913 *	0.793 *	−0.819 *	−0.542 *	−0.438 *	−0.920 *	−0.952 *	−0.515 *	−0.507 *	−0.141 *	−0.260 *
*p* value	0.001	>0.001	>0.001	>0.001	>0.001	>0.001	>0.001	0.014	0.053	>0.001	>0.001	0.020	0.023	0.554	0.268

* Correlation is significant at the 0.01 level (2-tailed).

## Data Availability

The original contributions presented in the study are included in the article, further inquiries can be directed to the corresponding authors.
